# Deployment of a *Vibrio cholerae* ordered transposon mutant library in a quorum-competent genetic background

**DOI:** 10.1128/mbio.00036-25

**Published:** 2025-02-25

**Authors:** Nkrumah A. Grant, Gracious Yoofi Donkor, Jordan Sontz, William Soto, Christopher M. Waters

**Affiliations:** 1 Department of Microbiology, University of Illinois Urbana-Champaign, Urbana, Illinois, USA; 2 Department of Microbiology, Genetics, and Immunology, Michigan State University, East Lansing, Michigan, USA; 3 BEACON Center for the Study of Evolution in Action, Michigan State University, East Lansing, Michigan, USA; 4 MSU College of Osteopathic Medicine, Michigan State University, East Lansing, Michigan, USA; 5 Department of Biology, College of William and Mary, Williamsburg, Virginia, USA; The Ohio State University, Columbus, Ohio, USA

**Keywords:** *Vibrio cholerae*, transposons, ordered mutant library, genetic competence

## Abstract

*Vibrio cholerae*, the causative agent of cholera, has sparked seven pandemics in recent centuries, with the current one being the most prolonged. *V. cholerae’s* pathogenesis hinges on its ability to switch between low- and high-cell-density gene regulatory states, enabling transmission between the host and the environment. Previously, a transposon mutant library for *V. cholerae* was created to support investigations aimed toward uncovering the genetic determinants of its pathogenesis. However, subsequent sequencing uncovered a mutation in the gene *luxO* of the parent strain, rendering mutants unable to exhibit high-cell-density behaviors. In this study, we used chitin-independent natural transformation to move transposon insertions from these low-cell-density mutants into a wild-type genomic background. Library transfer was aided by a novel gDNA extraction method we developed using thymol, which also showed high lysis specificity for *Vibrio*. The resulting Grant Library comprises 3,102 unique transposon mutants, covering 79.8% of *V. cholerae’s* open reading frames. Whole-genome sequencing of randomly selected mutants demonstrates 100% precision in transposon transfer to cognate genomic positions of the recipient strain in every strain analyzed. Notably, in no instance did the *luxO* mutation transfer into the wild-type background. Our research uncovered density-dependent epistasis in growth on inosine, an immunomodulatory metabolite secreted by gut bacteria that is implicated in enhancing gut barrier functions. Additionally, Grant Library mutants retain the plasmid that enables rapid, scarless genomic editing. In summary, the Grant Library reintroduces organismal-relevant genetic contexts absent in the low-cell-density-locked library equivalent.

Ordered transposon mutant libraries are essential tools for catalyzing research by providing access to null mutants of all non-essential genes. Such a library was previously generated for *Vibrio cholerae*, but whole-genome sequencing revealed that this library was made using a parent strain that is unable to exhibit cell-cell communication known as quorum sensing. Here, we utilize natural competence combined with a novel, high-throughput genomic DNA extraction method to regenerate the signaling incompetent *V. cholerae* ordered transposon mutant library in quorum-sensing-competent strain. Our library provides researchers with a powerful tool to understand *V. cholerae* biology within a genetic context that influences how it transitions from an environmentally benign organism to a disease-causing pathogen.

## INTRODUCTION

*Vibrio cholerae* is a human pathogen and the causative agent of the acute diarrheal disease cholera. While cholera is a global threat, the disease is endemic in developing countries, where it is estimated that there are between 1.3 and 4.0 million *V*. *cholerae* annual infections, resulting in 21,000–143,000 deaths (1, 2). Implementation of disease intervention strategies, including increasing access to clean drinking water, provision of adequate healthcare infrastructure, and educating citizens in communities at risk of disease spread, remains a top priority in the fight toward eradicating cholera. As such, the Global Task Force on Cholera Control was founded in 2017 to support efforts aimed at reducing the cholera burden by 90% before the year 2030. To date, there have been seven cholera pandemics, with the fifth and sixth pandemics caused by the Classical biotype and the seventh caused by the El Tor biotype (3)

The key to El Tor’s transition to a pathogen is that it is naturally competent, granting it the ability to take up DNA from the environment, which can be integrated into the genome by homologous recombination (4). Natural competence is a highly regulated process, and four environmental factors are key determinants for driving this process. These include nutrient limitation, extracellular signaling molecules, high-cell density, and the presence of chitin (5, 6), which together coordinate the expression of several regulators governing competence (7–10). Expressing the master regulator of competence TfoX can circumvent the chitin requirement for natural competence in *V. cholerae,* facilitating natural competence as a genetic tool for editing *Vibrio* spp. genomes (11, 12).

Our understanding of *V. cholerae’s* ecology and evolution has also benefited from transposon mutagenesis. An ordered mutant *V. cholerae* library was constructed in 2008. The ordered library, hereafter referred to as the Cameron library, consists of >3,100 mutants, each with a TnFGL3-based transposon insertion that confers kanamycin resistance. The Cameron library has provided a public resource enhancing *V. cholerae* genotypic and phenotypic investigations, supporting the identification of novel therapeutic targets for vaccine development (13), and increasing our understanding of the *V. cholerae* virulome (14, 15), among other discoveries (16, 17).

Although significant research advances have been made in our understanding of *V. cholerae* using the Cameron library, it was constructed in a presumed laboratory-acquired quorum-sensing (QS) mutant of *V. cholerae* (18). Quorum sensing, the process of cell-to-cell communication in bacteria, allows cells to undergo global changes in gene expression as the bacteria transition from low- to high-cell density. This transition can influence behaviors like host immunity escape (19), virulence factor production (20–22), biofilm formation (23), infectivity and environmental dissemination (24), and protection against phage predation (25). This transition is mediated by extracellular autoinducers (26, 27), which impact the activity of cytoplasmic regulators, including LuxO and HapR. The presumed wild-type *V. cholerae* strain, C6706, in which the Cameron library was constructed had acquired a mutation in *luxO, luxOG333S,* that locked this strain in the low-cell-density state. After sequence validating mutants from the 2008 library, we observed the *luxO*G333S mutation, indicating it was present in the parent strain from which the library was constructed. Thus, the 2008 ordered mutant library (28) is only partially representative of how *V. cholerae* senses and responds to its environment, and a new library in a quorum-sensing-competent strain would greatly enhance our understanding of *V. cholerae* ecology and evolution.

To overcome this limitation of the 2008 library, we use chitin-independent natural competence to regenerate the defective ordered *V. cholerae* library in a quorum-competent C6706 genomic background, hereafter referred to as the Grant Library. This approach offers several advantages over the chitin-dependent process, such as increased natural competence, leading to more efficient DNA uptake. Additionally, ectopic expression of the competence elements eliminates the need for chitinous shells in cultures, thereby saving time during mutant recovery and enhancing scalability. To streamline the transfer process, we developed a cost-effective gDNA extraction method using thymol (29). Compared to other methods, our approach is both economical ($0.64 per reaction vs $2.85 per reaction) and efficient, enabling up to 200 gDNA extractions in as little as 2.5 hours, in contrast to 25 reactions requiring >6 hours using conventional methods. Using our methods, we successfully regenerated the Cameron library with a 99% success rate, as indicated by growth on antibiotic selection growth media. Sequence validation of 23 randomly selected mutants from the Grant Library showed that in every mutant tested, the transposon recombined into our recipient strain with 100% precision. Furthermore, analysis of 93 mutants from the Grant Library never demonstrated the transfer of the *luxOG333S* mutation. Analysis of paired transposon mutants from each library provides evidence for density-dependent epistasis in several flagellar genes and growth on inosine—a metabolite secreted by gut bacteria—illustrating the utility of the Grant Library. Finally, we show that strains from the library generated in this work can undergo additional edits using chitin-independent natural competence. Taken together, the Grant Library will enable efforts toward understanding *V. cholerae* behaviors under transcriptional regulatory states that are representative of how this pathogen evolves and interfaces with its environment.

## MATERIALS AND METHODS

### Bacterial matings

To generate the library recipient strain used in this study, NG001, we mated wild-type *V. cholerae* C6706 with an *Escherichia coli* S17-lpir donor strain harboring plasmid pMMB-*tfoX-qstR* (generously provided by Ankur Dalia, Indiana University). In short, we mixed equal volumes of donor and recipient strains in the center of a Luria-Bertani agar plate, which we incubated at 37°C for 3 hours. Afterward, we added 1 mL of LB media containing chloramphenicol (for selection of plasmid in recipient strain) and polymyxin B (for counterselection of the donor *E. coli* strain) to the plate, washed, and collected the mating spot into a borosilicate glass culture tube, which we incubated overnight at 37°C with orbital shaking at 210 RPM.

### Media and buffers

#### Thymol cell lysis agent

We prepared thymol at a stock concentration of 350 mM by dissolving thymol crystals in dimethyl sulfoxide. Thymol stocks were made in 15 mL which we stored at room temperature on the lab bench until they were exhausted. We observed no differences in the lytic activity of thymol during storage.

#### gDNA extraction buffer

We prepared solutions of a gDNA extraction buffer in two 500 mL volumfe parts. We prepared solution one by mixing 80 mL of 1 M Tris (pH 8.0), 56 mL of 0.5 M EDTA (pH 8.0), 46.7 g NaCl, and 3 g sodium metabisulfite, which we stirred until dissolved. We then adjusted this solution to the final volume with dH_2_O and autoclaved it for 25 minutes. We prepared solution two, sodium acetate (2.5 M, pH 5.2), by dissolving 102.5 g of the salt in dH_2_O, which we sterilized using filtration. Prior to each extraction, we prepared a master mixture of solution one and solution two by mixing 21 and 31.5 mL for every 100 samples processed, respectively.

#### Minimal media with inosine

We prepared inosine at a stock concentration of 133 mM by mixing the compound in sterile dH_2_O. We then applied gentle heating and stirred the mixture until the solution was homogenous, which we then filter sterilized. We prepared 1× M9 minimal media amended with 0.1 mM CaCl_2_ and 2 mM MgSO_4_, to which we added inosine to a final concentration of 20 mM.

### Strain revival

We revived strains from the nonredundant Cameron library in microplate format to maintain parity between it and that produced in this work. The Cameron library was generously provided by the laboratories of Vic DiRita (plates: 2, 4–6, 8–10, 12–17, and 19–33), Michigan State University, and Bonnie Bassler (plates: 1, 3, 7, 11, 18, and 34), Princeton University. Briefly, microtiter plates from the Cameron library were slightly thawed, and 10 µL from each well was inoculated into a deep-well microplate containing 550 µL of selective media (Luria-Bertani broth + kanamycin [100 µg/mL]) in each of the 96 wells. We revived the Grant Library strain, NG001, in a 250 mL baffled flask containing 25 mL of selective media (Luria-Bertani broth + chloramphenicol [10 µg/mL]) to maintain plasmid *pmmB-tfoX-qstR* and 100 µg/mL isopropyl β-D-1-thiogalactopyranoside (IPTG) to induce natural competence. Unless noted otherwise, we grew all cultures overnight (18–24 h) at 37°C with orbital shaking at 210 RPM.

### Whole gDNA preparation

The non-redundant Cameron library comprised 34 96-well plates of *Vibrio cholerae* mutants. To extract genomic DNA from the Cameron library in a cost-efficient and high-throughput fashion, we developed an extraction method using a modified recipe of the gDNA extraction buffer developed by Mantel and Sweigart (accessed September 2022, at https://www.protocols.io/view/quick-amp-dirty-dna-extraction-4r3l287zql1y/v1). In brief, we pelleted cells from overnight cultures of the Cameron library by centrifuging the deep-well plates at 4°C for 20 minutes. We then removed ~500 µL of supernatant from each well, resuspended the cell pellet in 10 µL thymol (~35 mM final concentration), and incubated the plates at 64°C for 15 minutes. Thereafter, we added 500 µL of gDNA buffer to each well and incubated the plates at 64°C for an additional 45 minutes.

To clear culture supernatants of cellular debris, we transferred cell lysates from each well into an E-Z 96 Lysate Clearance Plate (https://www.omegabiotek.com/product/e-z-96-lysate-clearance-plate/). We then used a vacuum manifold to filter the supernatant through the plate, which we collected in a second deep-well plate containing 200 µL of 100% cold isopropanol per well. We then incubated the plates for 15 minutes at room temperature to precipitate genomic DNA. Afterward, we again used a vacuum manifold to filter the cell lysate containing our gDNA through an E-Z 96 DNA plate (https://www.omegabiotek.com/product/e-z-96-dna-plates/?cn-reloaded=1). According to the manufacturer, “E-Z 96 plates are silica glass fiber plates that can bind up to 50 µg of genomic DNA or 20 µg of plasmid DNA per prep.” After this initial filtration, we washed our bound DNA, in sequence, using 500 µL of 70% and 95% ice-cold ethanol. We then added 100 µL of 1× TE buffer to each well and eluted genomic DNA from the DNA plates into PCR plates by centrifugation (maximum speed, 1 minute).

### Chitin-independent natural competence for gDNA transfer

**Fig 1:**
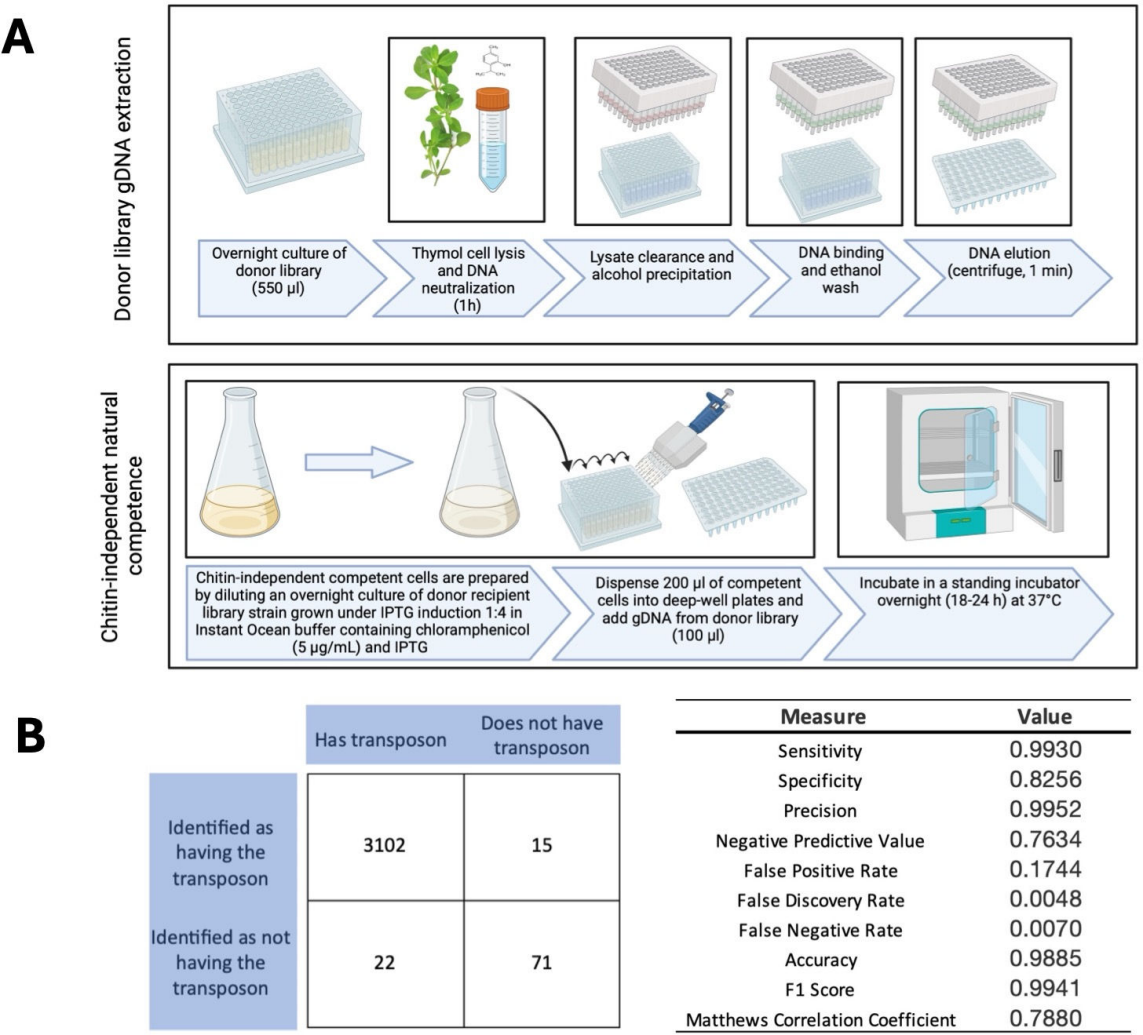
(A) Thymol-assisted gDNA extraction and chitin-independent natural transformation. Overnight cultures of the Cameron library were grown in 550 µL of selective media (LB + kanamycin). After overnight growth, the samples were centrifuged, the supernatant was aspirated, and the cell pellets were incubated for 1 hour at 64°C in a novel gDNA extraction buffer containing thymol. gDNA was then isolated from the clarified supernatant using an alcohol precipitation method, followed by resuspension of the gDNA in 1× TE buffer. In parallel, a quorum-competent wild-type *V. cholerae* strain carrying the plasmid conferring natural competence (pMMB-tfoX-qstR) was grown in selective media (LB + chloramphenicol) supplemented with IPTG. The next day, competent cells were diluted 1:4 in 0.5× Instant Ocean, to which chloramphenicol and IPTG were added. Subsequently, 200 µL of cells was dispensed into the wells of a deep-well 96-well plate and co-incubated with 100 µL of our gDNA extraction at 30°C for 18–24 hours. For a detailed description, refer Materials and Methods. (B) Analysis of library transfer. The outcomes of growth on selective media for each mutant were recorded after chitin-independent natural transformation of the Cameron library. These values were utilized as inputs to construct a 2 × 2 confusion matrix, categorizing the results as follows: true Positives (3,102 wells), false negatives (22 wells), true negatives (71 wells), and false positives (15 wells). The accompanying table presents the calculated measures, the values of which were computed using standard derivations.

After 18–24 hours of overnight growth, we diluted the library recipient strain (NG001) 1:4 in 0.5× instant ocean to which we added chloramphenicol (5 µg/mL) and IPTG (100 µg/mL). We then added 200 µL of these competent cells to each well of a deep-well 96-well plate and added the gDNA extracted from the Cameron library to a matched recipient well (final dilution 1:3). We then incubated the deep-well plates overnight in a standing incubator at 37℃. Following the overnight incubation, we added 400 µL of selective media (Luria-Bertani broth + chloramphenicol [5 µg/mL] + kanamycin [100 µg/mL]) to each well and incubated the plates at 37°C in a standing incubator for 48 hours (Fig. 1A). We determined transformation success by spot plating 3 µL of each well onto selective LB agar plates (Luria-Bertani agar + chloramphenicol [5 µg/mL] + kanamycin [100 µg/mL]), which we grew overnight at 37°C (Fig. S1).

### Grant Library transformation efficiency analysis

We created a comprehensive plate indices file (see Dataset S1 at https://github.com/NkrumahG/Grant-Library-Construction/blob/main/Grant-library-analysis/Plate_indices.txt) covering all possible wells within the Cameron library (P1A1 to P34D6; 3,210 wells). Using this file, we developed a script to compare it with a modified version of supplementary dataset 3 from reference (28) (see Dataset S2 at https://github.com/NkrumahG/Grant-Library-Construction/blob/main/Grant-library-analysis/LCD_locked_search.txt), which allowed us to identify empty wells (86) and those containing mutants (3,124). Our comparative analysis also flagged duplicate entries in supplementary dataset 3 of the Cameron library (see Dataset S3 at https://github.com/NkrumahG/Grant-Library-Construction/blob/main/Supplementary-datasets/Dataset%20S3.txt).

While constructing the Grant Library, we meticulously documented the outcomes of growth on selective media following the chitin-independent natural transformation of the Cameron library: true positives (3,102 wells), false negatives (22 wells were expected to grow but they did not grow), true negatives (71 blank wells that did not transfer), and false positives (15 blank wells that transferred unexpectedly). These values served as inputs for a binary confusion matrix (30), providing a comprehensive assessment.

### TnFGL3 transposon insertion site identification

#### Genome sequencing

We randomly selected 23 mutants from the Grant Library to identify the transposon insertion site. Strains were incubated overnight in 3 mL of LB + kanamycin (100 µg/mL), and DNA was extracted from 1 mL of culture using a Wizard Genomic DNA Purification Kit following the manufacturer’s instructions. DNA was rehydrated overnight at 4°C in 50 µL of TE buffer. Library preparation was performed by SeqCoast Genomics using an Illumina DNA Prep tagmentation kit and unique dual indexes. Samples were multiplexed and sequenced on the Illumina NextSeq2000 platform using a 300-cycle flow cell kit to produce 2 × 150 bp paired reads. Read demultiplexing, read trimming, and run analytics were performed using DRAGEN version 3.10.11, an on-board analysis software on the NextSeq2000. Raw sequencing data generated during this study are available in the following public Github repository (see Whole-Genome-Sequence-Analysis at https://github.com/NkrumahG/Grant-Library-Construction/tree/main/Whole-Genome-Sequence-Analysis).

#### Reference genomes

The reference genomes for *V. cholerae* strains used in this study were downloaded from NCBI GenBank. The chromosome accession numbers for chromosomes I and II are NZ_CP028827.1 (2,975,504 bp) and NZ_CP028828.1 (1,072,331 bp) for strain N16961 and NZ_CP046844 (3,019,938 bp) and NZ_CP046845 (1,070,359 bp) for strain C6706, respectively. The sequence map for plasmid pSC189 was downloaded from addgene (https://www.addgene.org/browse/sequence/36279/), and the sequence for pMMB-*tfoX-qstR* was provided by Ankur Dalia (Indiana University).

#### Mutation calling

We used consensus mode in *breseq* ([31], version 0.37) to align the paired-end reads for each of the sequenced mutants to the reference genomes. The computational resources used to perform *breseq* were provided by the Institute of Cyber-Enabled Research at Michigan State University.

#### Ortholog identification

We used Mauve ([32], snapshot _2015–02-25) to identify orthologs in whole genome sequences of C6706 and N16961. Each pair of chromosomes was aligned using the progressive Mauve method with default settings. In the supplemental material, we have included lists of all orthologous gene pairs in *V. cholerae* N16961 and C6706 (see Dataset S4 at https://github.com/NkrumahG/Grant-Library-Construction/blob/main/Supplementary-datasets/Dataset%20S4.xlsx) and the identity of genes unique to N16961 (see Dataset S5 at https://github.com/NkrumahG/Grant-Library-Construction/blob/main/Supplementary-datasets/Dataset%20S5.xlsx) and C6706 (see Dataset S6 at https://github.com/NkrumahG/Grant-Library-Construction/blob/main/Supplementary-datasets/Dataset%20S6.xlsx). Additionally, we used Geneious to extract, align, and visually display (Fig. S4 and S5) orthologous gene pairs for the transposon mutants sequenced in this study.

### LuxO gain-of-function mutation identification

Using supplementary dataset 3 provided for the Cameron library (28), we identified all potential transposon mutants located within a 50 kb range, both upstream and downstream of *luxO*. Subsequently, we developed a script that randomly picked 10 mutants from each side of *luxO*, using 5 kb windows. Following this selection, we cultured these mutants in selective media (LB + kanamycin, 100 µg/mL) for 24 hours under standard conditions.

After the incubation, we extracted gDNA using the thymol-based gDNA extraction method we developed to construct the Grant Library. The extracted genomic DNA was then diluted 1:2, which we used as a template for PCR amplification of the *luxO* gene. The *luxO* gene was amplified with Q5 polymerase using the following primers: forward primer 5′-GGCTATGCAACATAATCAATCTTG-3′ and reverse primer 5′-GCTTTGGTTGATCCATTCTCTCAT-3′. PCR conditions included an initial denaturation at 98°C for 2 minutes, followed by 29 cycles of denaturing at 98°C for 30 seconds, annealing at 63°C for 30 seconds, and extension at 72°C for 1 minute. A final extension step was performed at 72°C for 5 minutes.

Following the PCR amplification, we validated the amplification of *luxO* using gel electrophoresis. We then used the Qiaquick PCR purification system to purify our *luxO* PCR product. Sanger sequencing of the purified *luxO* allele was performed using the reverse primer 5′-TGCGATAGATGGTTGACGGG-3′ by the Roy J. Carver Biotechnology Center at the University of Illinois at Urbana-Champaign. We imported the Sanger Sequence read data into Geneious (33 ) and aligned it to the *luxO* gene on *V. cholerae* chromosome I (strain C6706). This allowed comprehensive analysis and visualization of our sequenced mutants, with particular focus on amino acid position 319. This position corresponds to the originally annotated site of the *luxO* gain-of-function mutation, characterized by an amino acid substitution from glycine to serine (*luxOG333S*) (34). However, subsequent studies have refined this annotation, identifying it as occurring at amino acid position 333 due to a previously miscalled start codon in the original genome annotation (35).

### Motility assay

We screened mutants with presumptive TnFLG3 transposon insertions in known motility genes. Strains were grown in a deep-welled 96-well plate overnight at 37°C in selective media (LB + kanamycin), and on the following day, we used toothpicks to manually stab each strain into motility plates containing LB and 0.35% agar. Plates were incubated at 37°C for 24 hours, and the motility zones were recorded with a gel imager. The area of the motility zones was measured computationally using the Hough Circle Transform package from the UCB vision plugin in Fiji (36).

### Inosine growth assay

We randomly selected five-paired 96-well plates from each of the Grant and Cameron et al. transposon mutant libraries and incubated them overnight in LB plus kanamycin (100 µg/mL). We also revived the parent strains used to construct each library in the same fashion. On the following day, we recorded mutants that did not grow overnight and then back diluted each well from the plates 1:100 into M9 minimal media supplemented with 20 mM inosine. We incubated all plates with linear shaking at 37°C. We monitored growth of the parent strains at an OD_600_ for 10 hours, with measurements taken at 5-minute intervals. For the paired library mutants, we measured the 10 hour endpoint optical density.

### Plasmid curing assay

We inoculated three cultures of NG001 from frozen glycerol stocks into LB media supplemented with chloramphenicol (10 µg/mL) and incubated them overnight. On the following day, we back diluted each culture 1,000-fold into either LB, LB media containing chloramphenicol (10 µg/mL), or LB containing IPTG (100 µM). For the duration of the experiment, we passaged each culture in the same media every 24 hours for 5 days (120 hours). In parallel, we serially diluted stationary phase cultures in 0.5× Instant Ocean and spot plated 3.0 µL from each dilution onto LB or LB agar containing chloramphenicol. The percentage of the population with the *pMMB-tfoX-qstR* plasmid (plasmid carriage) was calculated as a ratio of the dilutions where colonies grew on LB with chloramphenicol relative to growth on LB alone.

## RESULTS AND DISCUSSION

### Chitin-independent transfer of transposons from Cameron library strains into a quorum-competent *V. cholerae* genetic background is efficient

We hypothesized that the transposon insertions within the genomes of the quorum-incompetent *V. cholerae* strain generated in reference (28) could be transferred to a quorum-competent wild-type *V. cholerae* strain using natural competence. To test this, we extracted gDNA from each mutant in the Cameron library using an in-house method involving thymol lysis and alcohol precipitation. Subsequently, we co-incubated this gDNA with a wild-type *V. cholerae* strain (C6706) carrying a plasmid that expresses the natural competence master regulator, TfoX, upon induction with IPTG (gift of Ankur Dalia, Materials and Methods).

The Cameron library consists of 34 plates arrayed from P1A1 to P34D6, for a total of 3,210 possible wells containing transposon mutants. Upon examination of supplementary data table 3 for the Cameron library, we discovered that 86 wells were not listed, and some of the mutants were recorded multiple times. With these considerations, we expected growth for 3,124 unique transposon mutants after conducting our natural transformation protocol.

To verify the presence of transposon insertions in the Grant Library mutants, we spot-plated each mutant on LB medium supplemented with kanamycin. Of the 3,124 unique transposon mutants we expected to grow on the plates, 3,102 mutants (99.2%) exhibited growth (Fig. S1). None of the 22 missing mutants, which were expected to grow, showed growth in selective media during the overnight revival of Cameron library freezer stock. This suggests that these mutants might no longer be viable perhaps due to low culture density upon freezing or death resulting from repeated freeze-thaw cycling during long-term storage of the Cameron library. In the cases where we anticipated no growth, 71/86 (82.0%) did not grow on selective media.

To evaluate the performance of our library transfer approach, we utilized a confusion matrix (Fig. 1B). Our analysis showed that the Grant Library accurately achieved the expected density of mutants that should have transferred, with an F1 score of 0.9941 (scale 0–1, where 1 indicates perfect precision and recall). Moreover, our analysis showed that there was a very low frequency of false-positive transfer events, achieving a false discovery rate of 0.0048.

These results demonstrate the efficiency of using chitin-independent natural competence to transfer the transposons into a quorum-competent strain. The transference process was made possible through our in-house gDNA extraction method, involving thymol cell lysis and alcohol precipitation, affirming the suitability of this gDNA preparation for downstream molecular applications.

### All transposon mutants analyzed from the Cameron library recombine into the Grant Library parent genome with 100% precision

The frequency of genome edits by homologous recombination in *Vibrio* spp. is highest when there are at least 2 kB arms of homology flanking the genomic sequence being altered (12). Given this requirement, the observation that the recipient strains grew after incubation on selective media (Fig. S1) was suggestive that transposons correctly integrated into the homologous site of the recipient’s genome. To validate our supposition, we randomly selected 23 mutants, isolated gDNA, and sequenced whole genomes. Subsequently, we aligned the sequenced reads to four reference sequences using breseq (31). The reference sequences included *V. cholerae* chromosomes I and II, and the plasmid conferring natural competence. A fourth reference sequence (pSC189) containing sequences associated with the transposon was also included as a “genomic lure” to capture reads that mapped to the transposon. When examining the breseq output, we specifically looked for instances where new junctions were created between chromosomes I or II (depending on which chromosome the gene targeted for homologous recombination was located) and pSC189. Such a junction would indicate a transposon integration event. Figure S2 is an example breseq output from one such alignment, which reports the transposon insertion site in chromosome II, including the base pair position where it is located.

The parent strain of both the Cameron and Grant libraries is *V. cholerae* El Tor C6706. However, when the Cameron library was generated in 2008, transposon insertion sites were mapped to the reference genomes of *V. cholerae* El Tor strain N16961 because it was the only *V. cholerae* that had been sequenced for *Vibrio* spp. at that time (34). In this work, we mapped transposon insertion sites to the reference genomes of strain C6706. To identify whether transposon insertions were in the correct site of our recipient strains, we performed a whole genomic comparative analysis of *V. cholerae* N16961 and C6706. When we performed this analysis, we found that chromosomes I and II differ in size between strains and are not syntenic (Fig. S3). Out of the 2,649 genes annotated in our reference genomes for chromosome I, 2,558 (96.6%) of genes are shared between N16961 and C6706. Additionally, 21 genes (0.79%) are unique to N16961 and 70 (2.64%) to C6706. On chromosome II, 1,052 out of 1,064 annotated genes (98.9%) are shared between strains, where 10 (0.94%) and 2 (0.19%) of genes are unique to N16961 and C6706, respectively. Importantly, this analysis provided the genomic position for each ortholog of N16961 genes present in C6706 (see Dataset S4 at https://github.com/NkrumahG/Grant-Library-Construction/blob/main/Supplementary-datasets/Dataset%20S4.xlsx).

**Fig 2:**
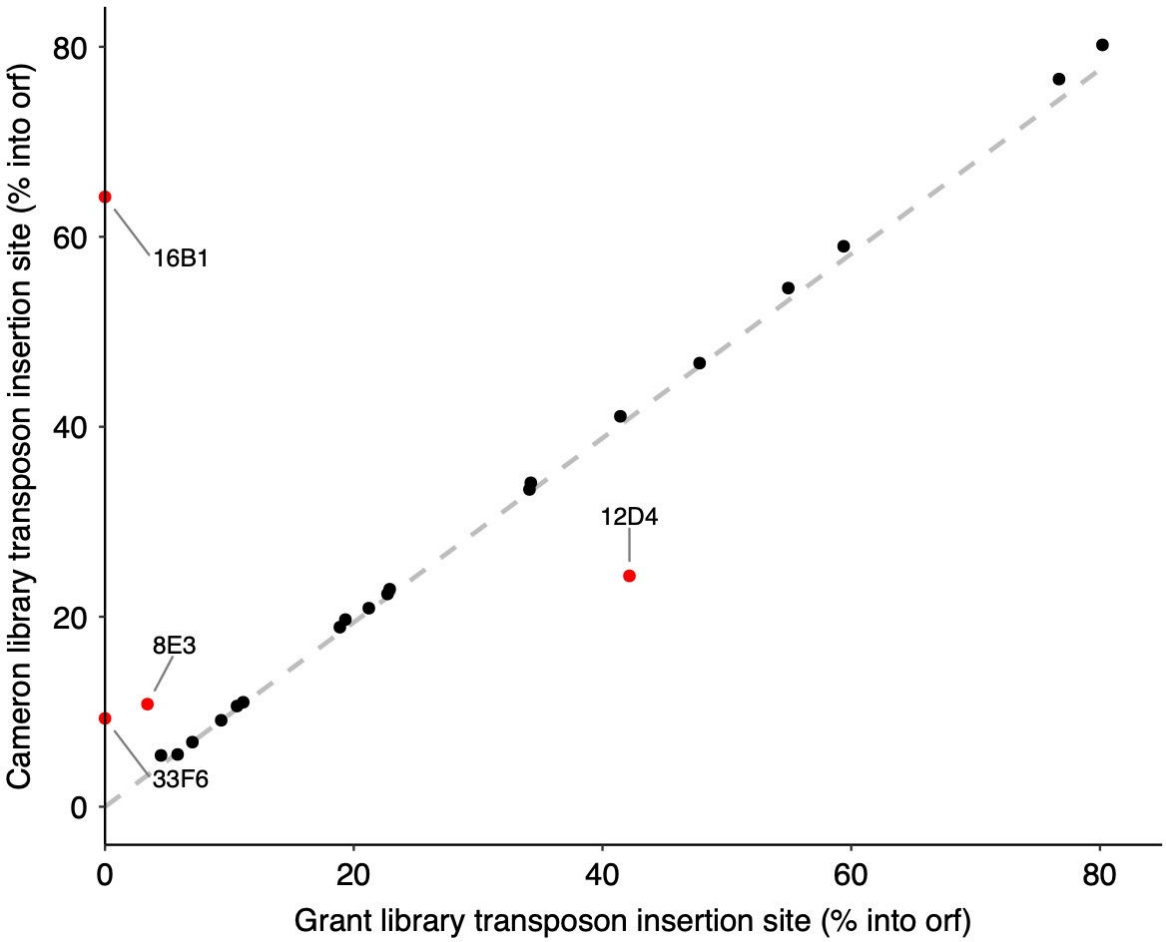
Correlation between transposon insertion sites in *V. cholerae* N16961 and C6706. Each point represents the transposon insertion site, calculated as percentage into the open reading frame. The values for strain N16961 were reported in supplementary dataset 3 of Cameron et al. (28), and in this work, they were calculated using the data provided in the annotation column of the breseq output (Fig. S2). The correlation analysis yields a strong positive correlation with Kendall’s coefficient *τ* = 0.752 (*n* = 23, and *P* << 0.0001), indicating a significant relationship between the insertion sites of the two strains.

We used the orthologous sites to analyze each transposon mutant we sequenced (Dataset S1; Fig. S4 and S5). Using the breseq annotation data, we calculated the position of the transposon within each gene as a percentage of base pairs into the open reading frame (ORF) of C6706. These position values were previously reported for each strain in the Cameron library strain N16961. To assess the relationship, we performed a linear regression of our sequenced C6706 insertions with the previously described N16961 from the Cameron library, expecting a perfect linear correlation. Although 19/23 mutants had an exact match, some of our values diverged from the line of best fit (Fig. 2), although there was a strong statistical correlation between the variables (Kendall’s coefficient *τ* = 0.752, *n* = 23, and *P* < 0.0001). Upon further investigation of this anomaly, we discovered two categories of false negatives. In category one (Fig. S5, panels A and B), we observed two cases where genes were annotated in N16961 but not in C6706. Consequently, these data points lacked a calculated value for the transposon insertion site in C6706 and thus fell on the *y*-axis. In the second category, two false negatives were attributed to a difference in the size of the gene orthologs between N16961 and C6706 (Fig. S5, panels C and D). This discrepancy resulted in a shift in the relative position reported for the transposon. After accounting for these deviations and correcting the data accordingly, we confirmed that in every mutant in the Grant Library we analyzed, the transposon inserted into the cognate position with 100% precision. However, it is important to note that we have not confirmed the correct transposon insertion of every mutant. Moreover, any mutants that were in the incorrect position of the Cameron library would also be present in the Grant Library. We, therefore, recommend confirming the location of any transposon insertion that will be used for future studies.

### Co-transformation of the *luxOG333S* gain-of-function mutation does not occur

Since we employed natural competence to transfer the transposon mutations, there is a possibility that the *luxOG333S* mutation from the Cameron library might have been co-transformed along with the transposon into the Grant Library. However, unlike the strong selection pressure for the transposon mutation, selection for the *luxO* mutation would be indirect and weaker, depending on whether the *luxO* mutation provides a fitness advantage to cells allowing them to outcompete wild-type cells during outgrowth in selective media.

In the 23 genome sequences analyzed above, none had the *luxOG333S* mutation. However, we did consider that the frequency of transfer of *luxO* mutation could be elevated in genes for which the transposon is within close genomic proximity to *luxO*. To test this, we isolated gDNA from 94 randomly sampled transposon mutants within a 50 kb window up- and downstream of *luxO* (see Dataset S7 at https://github.com/NkrumahG/Grant-Library-Construction/blob/main/Supplementary-datasets/Dataset%20S7.csv). We then PCR amplified *luxO* and performed Sanger sequencing. In no instance did the *luxOG333S* gain-of-function mutation transfer from the Cameron to the Grant Library parent strain (see Dataset S8 at https://github.com/NkrumahG/Grant-Library-Construction/blob/main/Supplementary-datasets/Dataset%20S8.pdf). This suggests that the proximity of the transposon mutations to *luxO* does not influence the transfer of the gain-of-function mutation; however, we do recommend sequencing *luxO* as a best practice when drawing comparisons between Cameron and Grant library strains.

### Natural competence in Grant Library strains is IPTG inducible

When generating the Grant Library, we supplemented the outgrowth media with chloramphenicol and kanamycin to select for the maintenance of the plasmid conferring IPTG-induced natural competence, pMMB-*tfoX-qstR*, and the TnFLG3 transposon, respectively. We reasoned that a library established with strains harboring this plasmid would be advantageous to the research community as it would facilitate additional genome edits using chitin-independent natural competence and homologous recombination upon induction with IPTG. In proof of concept for this idea, we randomly selected 10 mutants from the Grant Library and performed a knock-in experiment of a trimethoprim (tm) resistance gene in the pseudogene VC1087 as previously described (11). In short, we used thymol to isolate whole gDNA from a tm-resistant strain and added 16.2 µg of whole gDNA to our competent cells, which we prepared following our protocol described in Materials and Methods.

**Fig 3:**
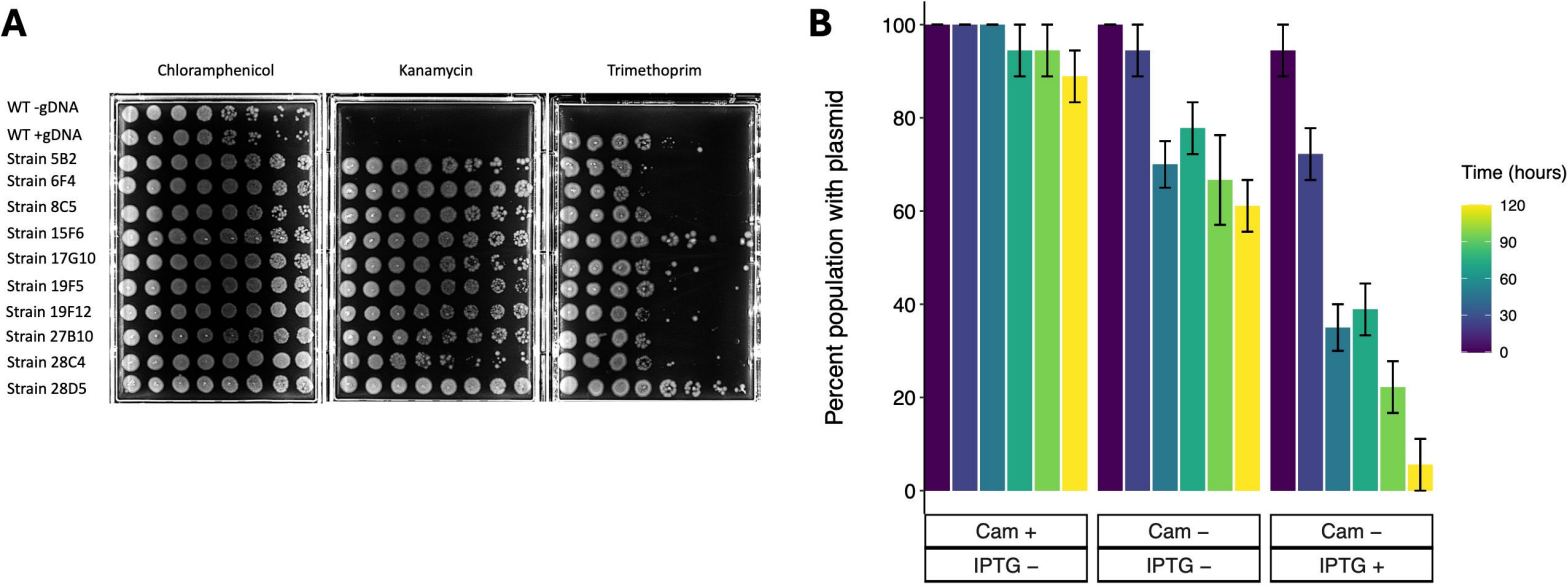
(A) Chromosomal integration of a trimethoprim-resistant gene in select mutants from the Grant Library using IPTG-induced natural competence. We induced natural competence in wild-type *V. cholerae* strain C6706 carrying plasmid pMMB-*tfoX-qstR* (NG001) and 10 randomly selected mutants from the Grant Library according to the procedure described in Materials and Methods. These strains were then co-incubated with gDNA extracted from a strain carrying a trimethoprim-resistant cassette and on the following day spotted on antibiotic selection media. All the strains carrying the plasmid grew on plates supplemented with chloramphenicol. On the plate with kanamycin, all Grant Library strains grew, validating that they have the transposon insertion. On the plate with trimethoprim, only the strains co-incubated with the trimethoprim-resistant gene grew, indicating that the Grant Library strains can undergo additional genomic editing. (B) Effects of media composition on plasmid loss in *V. cholerae*. We serially propagated cultures of the Grant Library parent strain in LB media with or without antibiotics or IPTG induction as indicated in the figure. The percentage of population with the plasmid conferring natural competence was calculated as a ratio of the dilutions where colonies grew on selective media (LB + chloramphenicol [Cam]) relative to non-selective media (LB). Error bars are the 95% confidence intervals on a sample size of *n* = 3 for each treatment.

After incubating our competent cells with gDNA overnight, we used spot plates to assess antibiotic phenotypes. All the randomly selected library mutants grew on LB plates supplemented with chloramphenicol, providing evidence that each has the plasmid conferring natural competence (Fig. 3A). Furthermore, all strains, less wild-type C6706, grew when plated on media supplemented with kanamycin, showing they each have a transposon insertion. When plated on LB supplemented with trimethoprim, all strains that underwent the knock-in experiment with the tm resistance gene grew, with colonies spotting between dilution factors 10^−3^ and 10^−7^. Taken together, all the library mutants we tested could undergo additional genome edits with overnight induction of natural competence.

### Plasmid conferring natural competence is readily cured from Grant Library strains with IPTG induction

Selecting for Grant Library transposon insertion mutants retaining the pMMB-*tfoX-qstR* plasmid allows for additional genomic edits to be constructed in each strain using chitin-independent natural competence. However, plasmid carriage might impose fitness costs that can vary between mutants due to genetic conflicts with each disrupted gene or complicate efforts to introduce alternative plasmids. Taking this into account, we sought to establish whether we could cure Grant Library strains of the plasmid. To this end, we serially passaged the recipient strain (NG001) carrying the plasmid in (i) LB media, (ii) LB supplemented with chloramphenicol and not IPTG, or (iii) LB without chloramphenicol but supplemented with IPTG. We hypothesized that amplifying plasmid carriage cost by inducing its expression in the absence of antibiotic selection (treatment three) would lead to rapid curing.

In agreement with our hypothesis, the number of cells carrying the plasmid decreased by ≈94% after 5 24-hour serial passages in LB media supplemented with IPTG alone (Fig. 3B). In contrast, plasmid carriage in cells passaged in LB alone or LB containing chloramphenicol decreased by ≈39% and ≈11%, respectively. These data show that the pMMB-*tfox-qstR* plasmid can be readily cured from Grant Library strains.

### Differential motility responses of Grant Library mutants reveal that unappreciated epistasis exists in Cameron library

*V. cholerae* exhibits high motility by virtue of a single polar flagellum, a characteristic that contributes significantly to its pathogenesis. *V. cholerae* isolated from active infections display increased motility compared to lab-grown strains (37) and demonstrated a heightened ability to colonize hosts (38). Moreover, motility allows *V. cholerae* to survive in its aquatic environment, enabling it to swim freely as planktonic cells or form biofilms in response to environmental stress. Furthermore, previous work has demonstrated the involvement of at least 40 genes in flagellum biosynthesis and motility, classified into temporally distinct classes (I through IV) (22, 39, 40). Notably, some of these genes are known to trigger an immune response (37).

Motility is an easily screened phenotype, and the functional consequences of null flagellar gene mutants are well established. Therefore, Cameron et al. (28) employed motility assays as one of the validation metrics for their constructed library. In this context, we also measured the motility of the Grant Library null flagellar gene mutants. By performing the assay using paired mutants from the Cameron and Grant libraries, we reasoned that we would foremost independently confirm the motility phenotypes reported by Cameron et al. (28). Furthermore, previous studies have indicated that genes in classes III and IV of the flagellum biosynthesis hierarchy exhibit cell density-dependent phenotypes (41). Thus, the motility assay would serve to provide supporting evidence for or against our hypothesis that quorum-sensing epistasis is a missing feature of the Cameron library.

**Fig 4:**
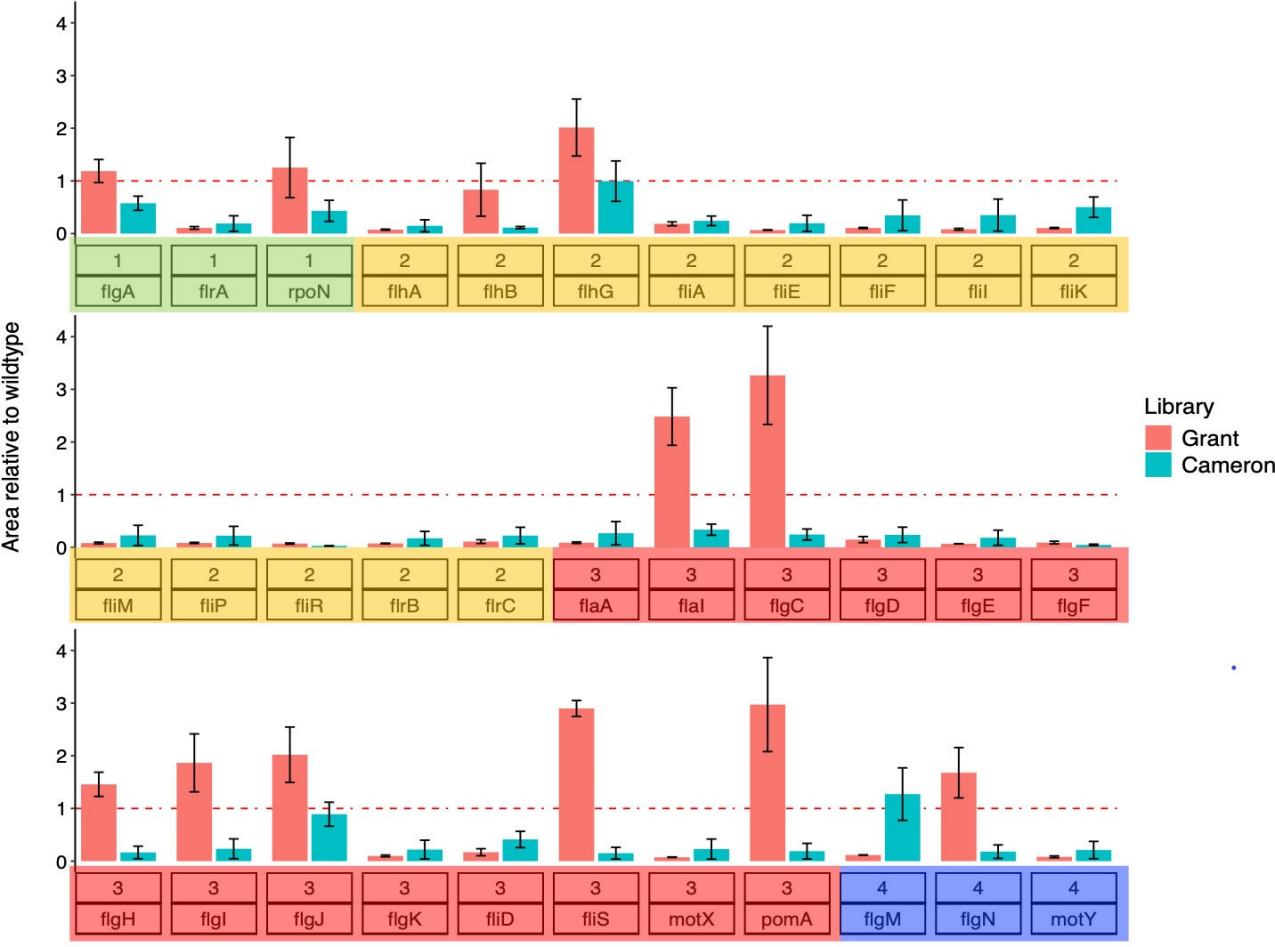
Motility behavior of flagellar mutants from the Grant and Cameron et al. libraries. Paired mutants with transposons in flagellar genes from both libraries were screened for motility in nutrient-rich plates containing 0.35% agar. The plates were imaged, and the distance moved by each mutant from the inoculation site was calculated using the procedure described in Materials and Methods. These areas were then normalized to a value relative to the wild-type strains for each library from three replicate assays in all but three cases (*flgM*, *flhB,* and *flgF*), which had two replicates each. The dashed red line is the normalization for the wild-type strains. Colored boxes highlight the class each gene belongs to in the flagellar biosynthesis gene cluster hierarchy.

Out of the 33 genes examined, we successfully replicated the motility phenotype reported for the Cameron library’s nonmotile mutants (Fig. 4). Additionally, we observed that both libraries exhibited nonmotile behavior for *flaA* (class III), a gene essential for the expression of the flagellar biosynthesis genes (Fig. 4). Furthermore, motility was also abolished for the σ54-dependent transcriptional regulator transposon mutants of *flrA* (class I) in both libraries, consistent with previous observations (42). When comparing motility within classes, paired null mutants from the Cameron and Grant libraries displayed similar movement on the plates for all classes, except for class III, where a statistically significant difference was observed (two-tailed *t*-test: *P* = 0.012). Additionally, two mutant pairs (*flgM* and *flgN*) within class IV biosynthesis genes exhibited opposite motility phenotypes. While these opposing effects were observed within the same class, the net effect of class IV genes canceled each other out in the statistical analysis, so this class comparison was insignificant. Taken together, our results suggest that quorum-sensing epistasis with motility genes may underscore the different motility outcomes, attributed to transcriptional state differences between the Cameron library’s low-cell-density-locked genomic background and a functional quorum-sensing pathway in the Grant Library.

### Growth on inosine is quorum dependent

Inosine is an essential nucleoside that plays an important role in purine biosynthesis and gene translation (43). Recent studies have demonstrated that various species of gut bacteria, including *Lactobacillus* (44) and *Bifidobacterium* (45), secrete inosine in significant quantities during growth. This production not only contributes to systemic immunomodulatory and protective functions but also enhances mucosal barrier functions within the gut (45). Additionally, inosine has been found to inhibit the expansion of pathogenic *Enterobacteriaceae* through PPARγ activation, further highlighting its importance in preventing gut dysbiosis (46).

In an unrelated screen analyzing for quorum-dependent growth effects using Phenotype Microarrays for Microorganisms (Biolog), we observed wild-type C6706 and a locked high-cell density (∆*luxO*) mutant exhibited enhanced growth when grown on inosine as the sole carbon source compared to a locked low-cell density mutant (∆*hapR*). As that the *luxOG333S* gain-of-function mutant locks the Cameron library into the low-cell density state, we reasoned screening of mutants in the Grant Library for growth on inosine could yield previously unappreciated epistatic effects between quorum sensing and central metabolism. Given *V. cholerae’s* manifestation of disease as an enteric pathogen—where inosine production is high—we sought to investigate whether these differences extended to other mutants from the Cameron and Grant transposon libraries. This exploration may shed light on the pathogenesis of *V. cholerae* and its interaction with inosine metabolism. Moreover, any significant differences between the transposon mutants would underscore the importance of using a library that does not mask phenotypic states relevant to *V*. *cholerae*’s ecology and evolution.

**Fig 5:**
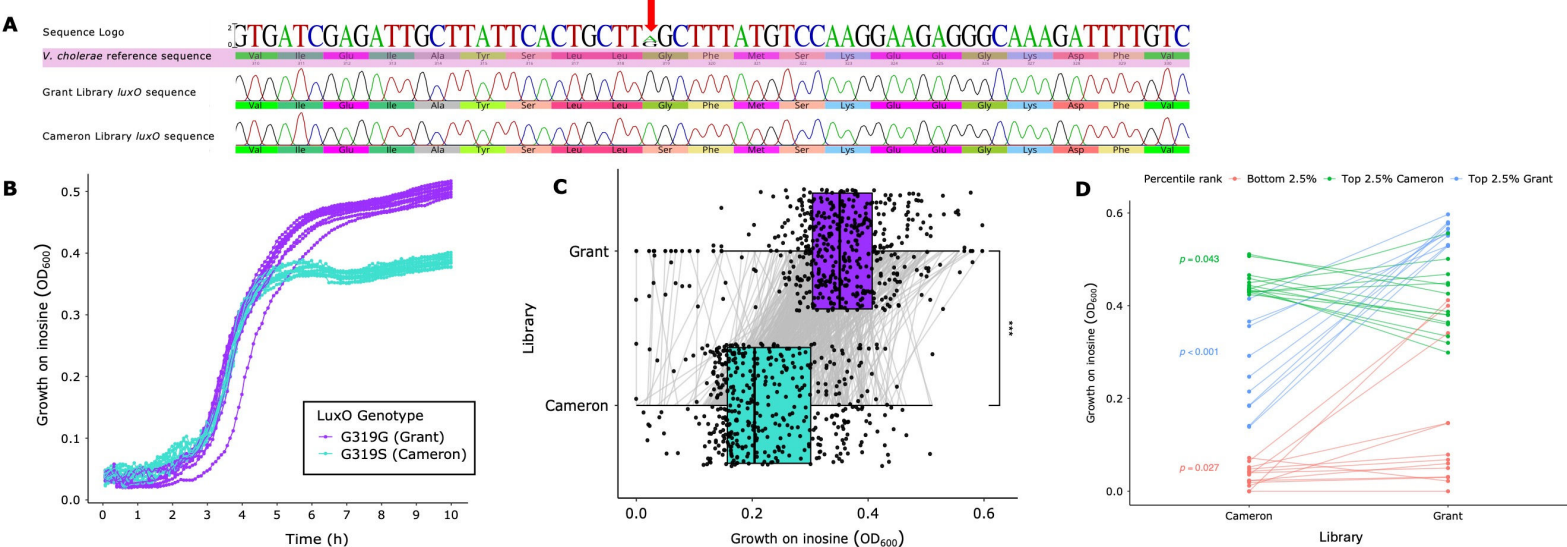
Genotypic and phenotypic characterization of Grant and Cameron library *V. cholerae* strains on inosine. (**A**) *luxO* sequence chromatogram for *V. cholerae* C6706 parent strains of Grant and Cameron transposon libraries. Red arrow shows the location of gain-of-function mutation resulting in a glycine-to-serine transition at amino acid position 333 in the Cameron library. (**B**) Growth of parent strains on inosine. Quorum-competent Grant Library strain grow to a higher OD (two-sample unpaired *t* = 29.34, *df* = 16, and *P* << 0.0001). (**C**) Boxplots of end-point optical density values for paired mutants from Grant and Cameron libraries grown on inosine. Each point represents a single mutant and the connecting lines the reaction norm for each mutant with respect to the library sampled from. Like the parent strains (**B**), Grant Library mutants grows to a higher OD than the Cameron et al. Library strains (two-sample unpaired *t* = −20.34, *df* = 904, and *P* << 0.0001). (**C**) Reaction norm for paired transposon mutants grown on inosine. Each point on the graph represents the 10-hour endpoint optical density for mutants from the Cameron and Grant libraries grown on inosine, with lines connecting paired mutants. We identified outliers by focusing on the top and bottom 2.5% of the data, calculated using the mean and standard deviation for each library. Relationships are plotted for points that deviated two standard deviations from each mean.

We began our investigation by Sanger sequencing the *lux*O locus of the parent strains for the Cameron and Grant libraries. The sequence chromatograms (Fig. 5A) validated the glycine-to-serine gain-of-function mutation at amino acid position 333 in the Cameron library parental strain. We then grew replicate cultures of the parent strains in M9 minimal media supplemented with 20 mM inosine (see Dataset S9 at https://github.com/NkrumahG/Grant-Library-Construction/blob/main/Supplementary-datasets/Dataset%20S9.csv). There was no significant difference in the growth rates of the parent strains. However, the Grant Library parent strain bearing the ancestral *luxO* allele grew to an endpoint optical density that was on average ≈23% higher than the Cameron library parent strain (Fig. 5B), and the relationship was statistically significant (two-sample unpaired *t* = 29.34, *df* = 16, and *P* < 0.0001).

We then randomly selected and revived five-paired plates from the Cameron and Grant libraries and grew them in inosine for 24 hours (see Dataset S10 at https://github.com/NkrumahG/Grant-Library-Construction/blob/main/Supplementary-datasets/Dataset%20S10.csv). In agreement with the experiments performed with the parent strains, the Grant Library transposon mutants grew to an endpoint optical density that was 35% higher than the Cameron et al. library (Fig. 5C), and the relationship was statistically significant (two-sample unpaired *t* = −20.34, *df* = 904, and *P* < 0.0001).

Reaction norms describe the phenotypic expression of a single genotype across an environmental gradient (47). Given our experimental design and the isogenicity of the Cameron and Grant strains, we adopted this method to visualize deviations between paired library mutants grown on inosine (Fig. 5C, gray lines). We focused our attention on mutants whose growth on inosine was above and below two standard deviations (top and bottom 2.5% of the data, respectively) away from the mean for each library (Fig. 5D).

The data show strong evidence of quorum-dependent epistasis. When we looked at the bottom 2.5% of all the data, 7 of the 13 transposon mutants were shared between libraries and each failed to grow on inosine (Table S1). The functional roles of the associated genes included amino acid biosynthesis, energy metabolism, and regulatory functions. That these mutants failed to grow independent of the *luxO* genetic background likely reflects the essentiality of these genes when inosine is used as the sole carbon source. For the remaining six genes, five grew better on inosine in the Grant Library genomic background and one marginally less, though the average growth on inosine for the mutants from each library was statistically significant (two-sample paired *t* = −2.52, *df* = 12, and *P* = 0.027).

Interestingly, when examining the top 2.5% of the data for the Grant Library (Fig. 5D), there was strong anticorrelation with paired mutants from the Cameron library (two-sample paired *t* = −8.90, *df* = 10, and *P* < 0.001). That is, where growth was high for a mutant in one library, its growth was lower in the opposite. For the top 2.5% of mutants with the highest growth on inosine in the Cameron library, the paired response for Grant Library mutants was more varied, and the average difference was only marginally significant (two-sample paired *t =* 2.25, *df* = 13, and *P* = 0.043). In stark contrast to the bottom 2.5% of genes, only one transposon mutant (gene disruption of fusA-2) was shared within the top 2.5% of each data set, and there was more variation in the functional roles of all called mutants (Table S2). We hypothesize that the nonlinearity in the top 2.5% is due to epistasis and the differential expression of genes at low and high cell densities in *V. cholerae*.

Collectively, our inosine findings show that ecologically relevant phenotypes can be masked for *V. cholerae* in quorum-dependent contexts and are supported by several other research groups who as well have observed nuanced phenotypes in the *luxOG333S* genetic background.

### Conclusion

Bacteria employ QS to regulate gene expression based on population density. In the case of *V. cholerae*, the etiological agent of cholera, QS controls various phenotypes as the population transitions between low- and high-cell density. Researchers previously constructed an ordered mutant library, systematically disrupting every non-essential gene in *V. cholerae* with a transposon insertion. However, unbeknownst to them, this library was created in a strain with a mutation that rendered the mutants incapable of transitioning between low- and high-cell density.

In this study, we successfully transferred transposon insertions from these *V. cholerae* non-redundant ordered mutants into a wild-type genetic background using chitin-independent natural transformation. The resulting Grant Library comprises 3,102 mutants, covering approximately 79.8% of the ORFs annotated in *V. cholerae*.

In addition to encoding a functional quorum-sensing system, another notable advantage of the Grant Library is that we selected for the transposon insertion and the plasmid that confers IPTG-inducible natural competence during outgrowth in selective media. Accordingly, upon induction with IPTG, mutants from the Grant Library can undergo additional genome edits when co-incubated with gDNA. Furthermore, growth in the absence of selection leads to rapid curing of the competence plasmid if it is not needed. These features make the Grant Library an invaluable resource for studying pleiotropic and epistatic genetic interactions in *V. cholerae*. Indeed, the nonlinearity between paired mutants when grown on inosine demonstrates this utility. Ongoing work in the Grant lab involves utilizing the two transposon libraries described in this work, and a third high-cell-density-locked library we are generating, to deconstruct gene × transcriptional state × environmental interactions, including synthetically lethal genetic combinations. Such information will be invaluable toward generating new therapeutic options to treat *V. cholerae* infections.

Construction of the Grant Library was aided by a novel, in-house gDNA extraction method we developed based on thymol cell lysis. Our use of thymol allowed the extraction of gDNA in a highly efficient and economical way. Indeed, N.A.G. has performed as many as 576 manual gDNA extractions in a single workday. Additionally, at the concentrations used in our studies, gDNA extracted with thymol does not negatively impact its use in other downstream applications, including PCR amplification and whole genome and Sanger sequencing. Given thymol’s cell-killing efficacy in *Vibrio*, we tested the treatment on several other biological specimens, including bacteria in gram-negative and -positive staining classes and yeast. Among our test strains, gDNA yield was highest for *V. cholerae* (~22 ng/µL), although most strains yielded some gDNA (2–8 ng/µL). That *Vibrio* is highly susceptible to thymol treatment is an interesting avenue for further research as its understanding can provide additional insight into its antibacterial activity, potentially contributing to novel disease control strategies. In summary, the Grant Library complements the non-redundant library made in the quorum-incompetent strain and the methods used to construct it, which is a valuable approach toward understanding *V. cholerae* evolutionary genetics.

## Data Availability

All data generated and analysis scripts used in this work are available in the GitHub repository at https://github.com/NkrumahG/Grant-Library-Construction.

## SUPPLEMENTAL MATERIAL

Supplemental material mbio.00036-25-s0001.docx Fig. S1-S5; Tables S1 and S2; information regarding supplemental data sets.

ASM does not own the copyrights to Supplemental Material that may be linked to, or accessed through, an article. The authors have granted ASM a non-exclusive, world-wide license to publish the Supplemental Material files. Please contact the corresponding author directly for reuse.
