# Supplementary material for: Deployment of a Vibrio cholerae ordered transposon mutant library in a quorum-competent genetic background
Source: mBio. 2025 Feb 25;16(4):e00036-25. doi: 10.1128/mbio.00036-25 (PMC11980543; doi:10.1128/mbio.00036-25)
Supplement: Supplemental material — Fig. S1-S5; Tables S1 and S2; information regarding supplemental data sets. [file mbio.00036-25-s0001.docx]

**Supplementary information text**

**
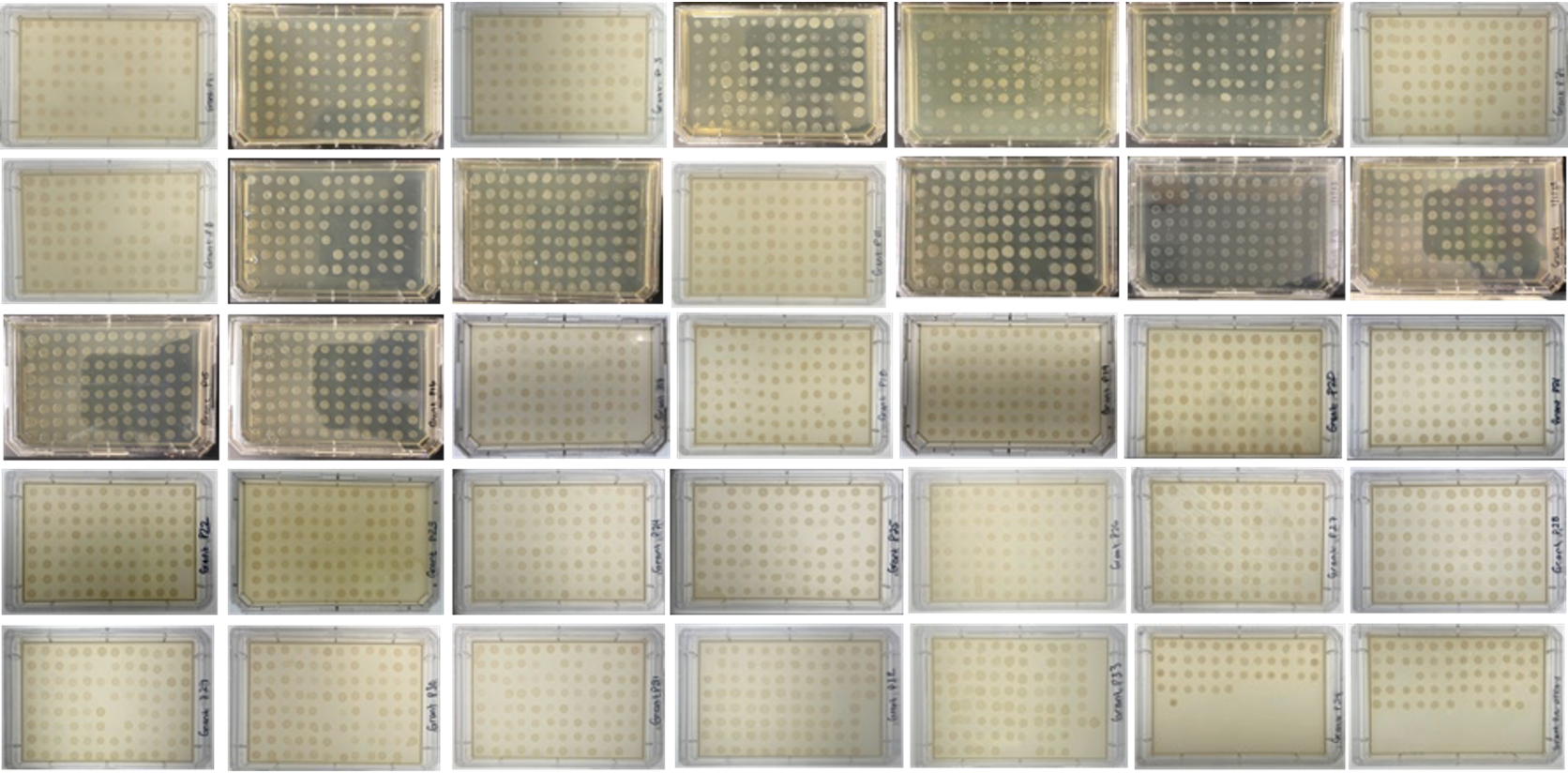
**

Figure S1. Growth of Grant library transposon mutants on antibiotic selective media. Each mutant was spot plated on LB agar + kanamycin and chloramphenicol to select for mutants with the transposon insertion and the plasmid conferring natural competence (pMMB-*tfoX*-*qstR*), respectively. Plates were incubated overnight, and the growth of each strain was recorded on the following day.


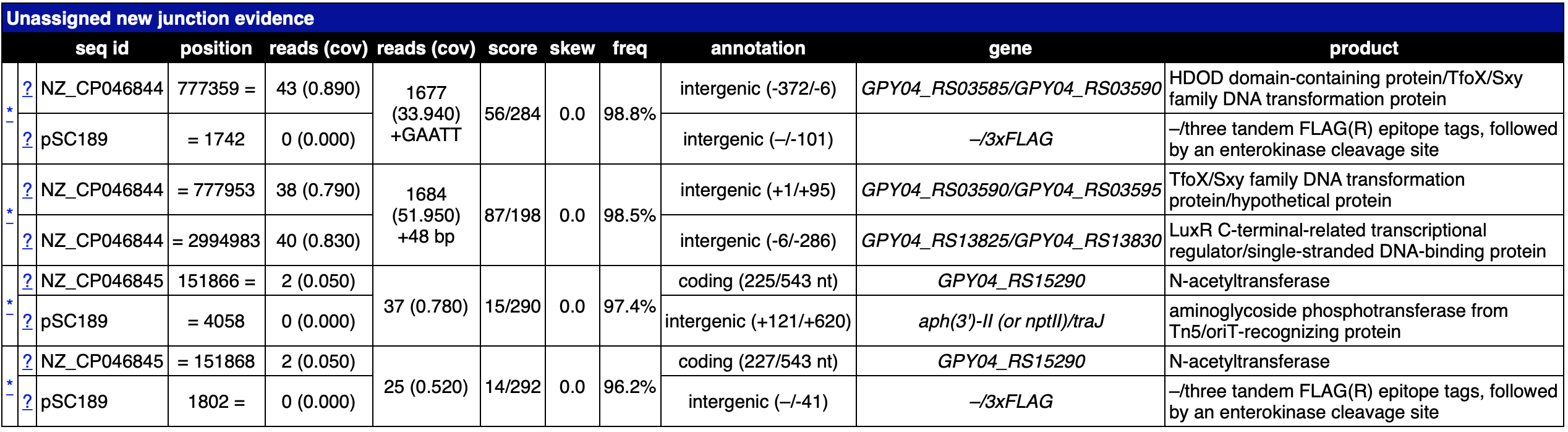

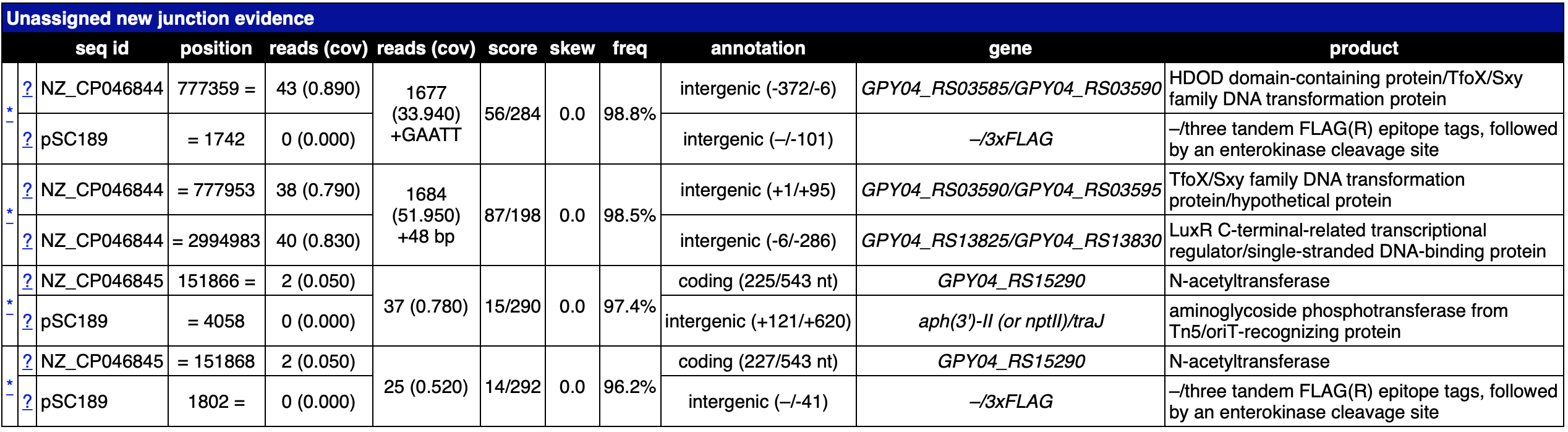


Figure S2. Representative sample of breseq output showing new junction evidence supporting transposon insertion. The figure shows a portion of the summary.html file generated for a breseq analysis run. In this example, a new junction has been called between chromosome II (NZ_CP046845) of *V. cholerae* strain C6706 and the plasmid (pSC189) containing representative sequences of the transposon. Insertions have left and right boundaries, where the chromosome and sequence meet. The boundaries are marked with an asterisk. The annotation column shows the position of the transposon within the gene (GPY04_RS15290), which we converted to a value called “percent into open reading frame.” In this example, that value is 41.43% ((225/543) * 100)). This value was calculated for all strains sequenced in this study.

Figure S3. Genomic comparative analysis of *V. cholerae* N16961 and C6706 for chromosome I (A) and chromosome II (B). Genbank files for whole-genome sequences of chromosomes I and II of both strains were downloaded from NCBI. The analysis involved a comparative examination of genetic variations and conservation patterns between the two strains. *V. cholerae* strain N16961 is shown with a gray background and C6706 a white one. Genome segments are shown as colored colinear blocks centered around the “positive” and “negative” strands of each chromosome. Lines connecting the segments show their orthologous alignments between the strains, including inversions. Panel C is a Venn diagram summarizing the relationship of genes for each strain and chromosome. The data underlying the illustration in panel C, including the genomic position of orthologous gene pairs, is provided in the Supplementary Information (Datasets S2–S4).

Figure S4 (panels A–S)

**Mutant 1D3**

**Mutant 2C1**

**Mutant 2D5**

**Mutant 4B2**

**Mutant 6H11**

**Mutant 9E2**

**Mutant 10H8**

**Mutant 13H5**

**Mutant 19H8**

**Mutant 21C6**

**Mutant 24B2**

**Mutant 24D2**

**Mutant 24G4**

**Mutant 28E11**

**Mutant 29E1**

**Mutant 31A10**

**Mutant 31E11**

**Mutant 31F3**

**Mutant 33C7**

Figure S4: Comparison of Transposon Insertions between *V. cholerae* N16961 and C6706. Each panel (A–S) displays the alignment for genes that lie on the regression line (Fig. 2) in the indicated samples. Transposon insertion sites were determined from whole-genome sequence analysis of C6706 (this work) and for N16961 from Cameron et al. (2008). These sites are highlighted above the representation of each gene sequence in blue, and the names of each strain and the identity of each gene are shown within the colored blocks.

Figure S5 (panels A–D)

**Mutant 16B1**

**Mutant 33F6**

**Mutant 8E3**

**Mutant 12D4**

Figure S5: Comparison of Transposon Insertions between *V. cholerae* N16961 and C6706. Each panel (A–D) displays an alignment of genes whose points deviated from the regression line (Fig. 2) in the indicated samples. Panels A and B illustrate category one false negatives, where transposon insertions are mapped to genes annotated in *V. cholerae* N16961 but not in C6706. These correspond to points that fall on the y-axis in Fig. 2. Panels C and D illustrate category two false negatives, where each gene pair aligns with > 99.8% homology between N16961 and C6706, but they differ in size, accounting for the divergence seen for those points in Fig. 2.

Table S1. Gene identities for the bottom 2.5% of mutants from the Grant or Cameron libraries grown in minimal media supplemented with 20 mM inosine as the carbon source. Colors indicate whether a mutant was present (green) or absent (red) in this subset of data.

Table S2. Gene identities for the top 2.5% of mutants from the Grant or Cameron libraries grown in minimal media supplemented with 20 mM inosine as the carbon source. Colors indicate whether a mutant was present (green) or absent (red) in this subset of data.

All supplementary Datasets S1-S10 can be found here: [https://github.com/NkrumahG/Grant-Library-Construction](https://urldefense.com/v3/__https:/github.com/NkrumahG/Grant-Library-Construction__;!!HXCxUKc!1pz2QE9X-iBVGYPfSN77k8rxPQk8dgy5mDMwrQX3hBKBqqT1UvZOyYxQTGT79FOGa_c9SXzFDlvFGRew7SNsLw$).

Dataset S1. Plate indices file

Dataset S2. Modified version of transposon library mutant dataset presented in Cameron et al., 2008.

Dataset S3. List of genes duplicated in the Cameron et al., 2008 transposon library mutant dataset.

Dataset S4. List of gene ortholog pairs present in *Vibrio cholerae* strains N16961 and C6706.

Dataset S5. List of genes unique to *Vibrio cholerae* strain N16961.

Dataset S6. List of genes unique to *Vibrio cholerae* strain C6706.

Dataset S7. List of transposon insertion mutants within a 50 kb window upstream and downstream of *luxO*.

Dataset S8. Sanger sequence chromatograms for 94 transposon mutants sequenced in 50 kb window upstream and downstream of *luxO*.

Dataset S9. OD_600_ readings over time monitoring growth of Grant and Cameron et al. parent strains in minimal media supplemented with 20 mM inosine.

Dataset S10. Endpoint OD_600_ readings for 5-paired plates from Grant and Cameron et al. transposon mutant libraries grown in minimal media supplemented with 20 mM inosine.

Table S1. Gene identities for the bottom 2.5% of mutants from the Grant or Cameron libraries grown in minimal media supplemented with 20 mM inosine as the carbon source.

Table S2. Gene identities for the top 2.5% of mutants from the Grant or Cameron libraries grown in minimal media supplemented with 20 mM inosine as the carbon source.
